# Cercopidae spittle-bugs (Hemiptera, Cicadomorpha) of Madagascar: a new species of *Bourgoinrana* and revision of the *Locris* species

**DOI:** 10.3897/zookeys.1023.58733

**Published:** 2021-03-10

**Authors:** Emilien Bouteille, Maxime Le Cesne, Adeline Soulier-Perkins

**Affiliations:** 1 Mécanismes adaptatifs et évolution (MECADEV), Muséum national d’histoire naturelle, CNRS, 57 rue Cuvier, CP 50, 75005 Paris, France Mécanismes adaptatifs et évolution (MECADEV), Muséum national d’histoire naturelle, CNRS Paris France

**Keywords:** Afrotropical, Cercopoidea, key, male genitalia, taxonomy

## Abstract

The *Locris* species and subspecies from Madagascar are revised and a new combination is proposed: Locris johannae var. nigrolimbata Lallemand, 1910 to *L. nigrolimbata***comb. nov**. Illustrations and description of male terminalia are given for the first time for the three *Locris* species and an identification key is provided. A new species of the Malagasy endemic genus *Bourgoinrana* Soulier-Perkins, 2012 is described: *B. beondrokaensis* Le Cesne & Soulier-Perkins **sp. nov**. An updated identification key to the species of *Bourgoinrana* is provided.

## Introduction

Hemiptera are one of the most diverse groups of insects (Bartlett et al. 2018). They are mainly distinguished by a biting-sucking mouth apparatus. Many of them are phytophagous, but some feed on other liquids such as from animals or fungi. Despite their great diversity, some families, such as the CercopidaeLeach (1815), remain little studied. Hamilton (2014) removed two genera: *Ambonga* Melichar, 1915 and *Pseudomachaerota* Melichar, 1915 from the Malagasy Cercopidae, leaving this family with nine genera known for the island. Six of these genera are endemic: *Alluaudensia* Lallemand, 1920, *Amberana* Distant, 1908, *Bourgoinrana* Soulier-Perkins, 2012, *Nesaulax* Jacobi, 1917, *Paramioscarta* Lallemand, 1949 and *Pogonorhinella* Schmidt, 1910. The remaining three genera *Literna* Stål, 1866, *Locris* Stål, 1866 and *Rhinaulax* Amyot & Serville, 1843 are present as well on the African continent. *Locris* is one of the largest genera of Cercopidae, with 87 known species, according to COOL (Soulier-Perkins 2020). It is widespread throughout tropical and South Africa as well as in Madagascar. A revision of Malagasy *Locris* species is presented here with male genitalia drawings and photos of habitus, and a new species of *Bourgoinrana* is described and the key to the species updated.

## Material and methods

The abdomen of each specimen examined was cut off and cleared for one hour in hot (85 °C) 10% KOH. Dissections and cleaning of genital structures were performed in distilled water. If needed, a few drops of blue paragon for dying the ectodermic genital ducts were added for a few minutes. Observations were done in glycerol using a Leica microscope (MZ16). Drawings were produced using a camera lucida attached to the microscope and finalised with Illustrator CS6 (Adobe Inc. 2012). Photos of the habitus were taken using a Canon EOS 6D with a Macro Lens Canon EF 100 mm f/2.8, viewed on computer with the software Canon EOS utility and then assembled with the software Helicon Focus 6. Terms used for the male genitalia are those of Soulier-Perkins and Kunz (2012). Qgis 3.10 (2020) was used to draw the distribution map.

### Abbreviations

**CAS**California Academy of Sciences, San Francisco, USA;

**MIIZ** Muzeum i Instytut Zoologii, Warsaw, Poland;

**MNHN**Muséum national d’Histoire naturelle, Paris, France;

**MRAC**Musée royal de l’Afrique central, Tervuren, Belgium;

**RIScNB**Institut Royal des Sciences Naturelles de Belgique, Bruxelles, Belgium.

## Taxonomy

### Locris

Taxon classificationAnimaliaHemipteraCercopidae

Stål, 1866

A49DAF05-C29D-5EA3-AB2F-A4681CDC0CEF

#### Type species.

*Locris rubra* (Fabricius, 1794)

The genus *Locris* Stål, 1866 was largely studied by Lallemand (1949) on the basis of morphological characters that are not all completely consistent in all species according to our observations. These characters are as follows: postclypeus rounded with a medio-longitudinal carina and transverse ridges, when observed laterally; it can be, either rounded (*L. rubra* (Fabricius, 1794)), angular (*L. vestigans* Jacobi, 1904) or protruding (*L. schmidti* Jacobi, 1910). Rostrum is short and barely extends to base of median trochanters. Antennae are short and their length equals diameter of eyes. Vertex is broader than long and its length equals half width of pronotum. Ocelli are much closer to each other than to compound eyes and are medium to small in size, except in *L. atra* Lallemand, 1923 where they are very large (Lallemand 1949). Pronotum is large, with a usually straight posterior margin but weakly indented in some species like *L. maculata* (Fabricius, 1781) according to Lallemand (1949) and may have a more or less distinct carina in middle. Scutellum is as long as wide and has three dimples, two small ones on anterior margin and one large centred. Tegmina are about 3 times as long as wide, cubital and median veins are fused from base to middle of tegmen, apical veins network is relatively dense. A spine is present on posterior tibiae. In genitalia, males have thin subgenital plates that look like a filament (Lallemand 1949) curved up or downward; they are wide and sometimes silky at base.

In describing the genus *Locris*Lallemand (1949) listed a series of exceptions in order to include more species in the genus. As a result, our view is that now the homogeneity of the genus and its taxonomic unity is questionable. However, the aim of our work here is not to revise the entire genus (which is present in Madagascar and the whole of Africa except for the northern countries Egypt, Tunisia, Libya, Algeria and Morocco) but to provide a clear identification for the few species and subspecies of *Locris* present in Madagascar.

### Locris bipunctata

Taxon classificationAnimaliaHemipteraCercopidae

(Signoret, 1860)

C8A5D463-9787-5A1C-B267-B1B54DE2FB9B

Figures 1 , 2 , 3

Monecphora bipunctata Signoret, 1860: 182 (original description)
Locris bipunctata : Stål 1866: 60 (transfer)
Locris bipunctata var. atra Lallemand, 1950: 94

#### Note.

**Figure 1.**
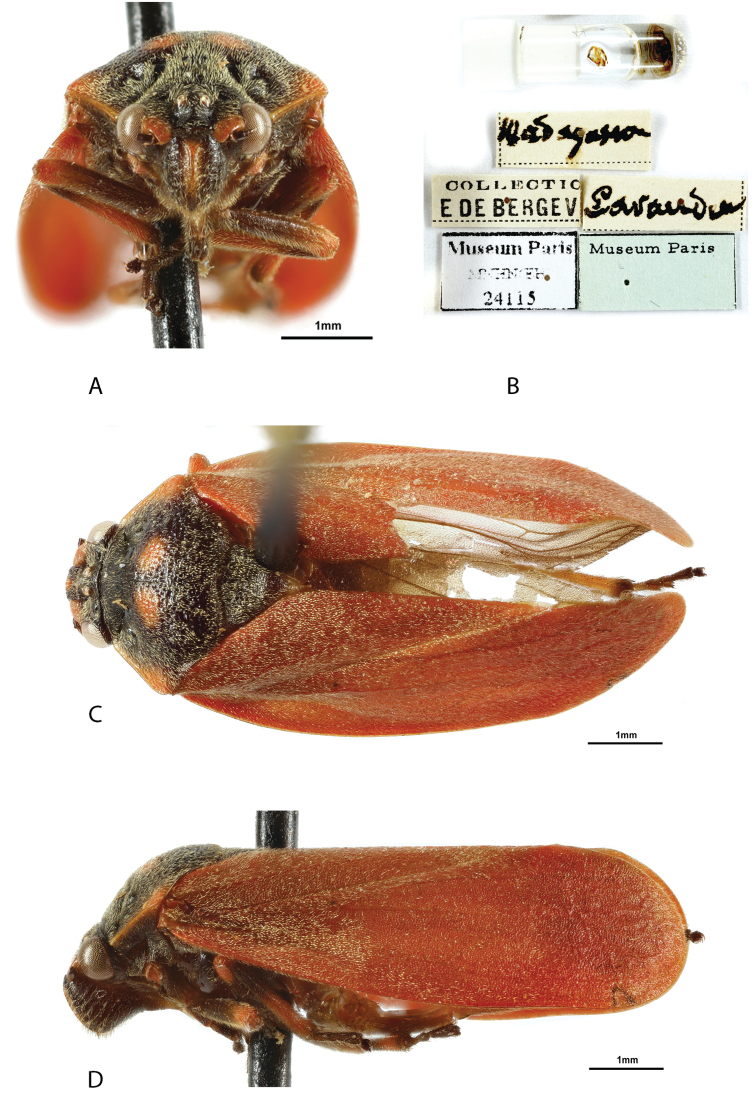
*Locris bipunctata bipunctata* (Signoret), male **A** frontal view **B** labels **C** dorsal view **D** lateral view.

**Figure 2.**
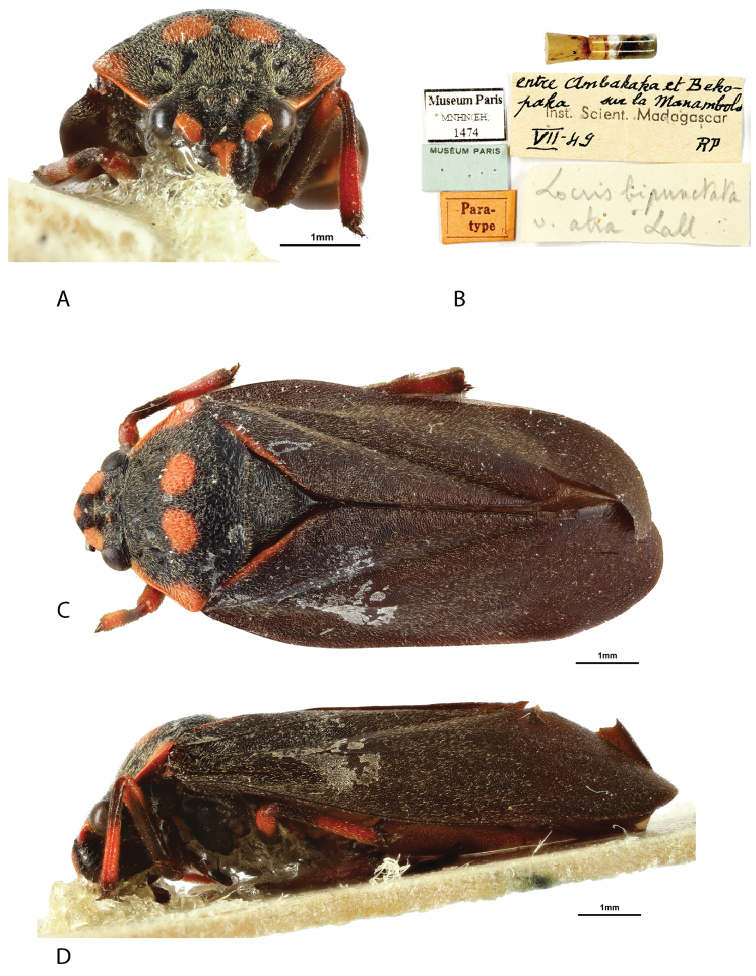
*Locris bipunctata atra* (Lallemand), male paratype **A** frontal view **B** labels **C** dorsal view **D** lateral view.

**Figure 3.**
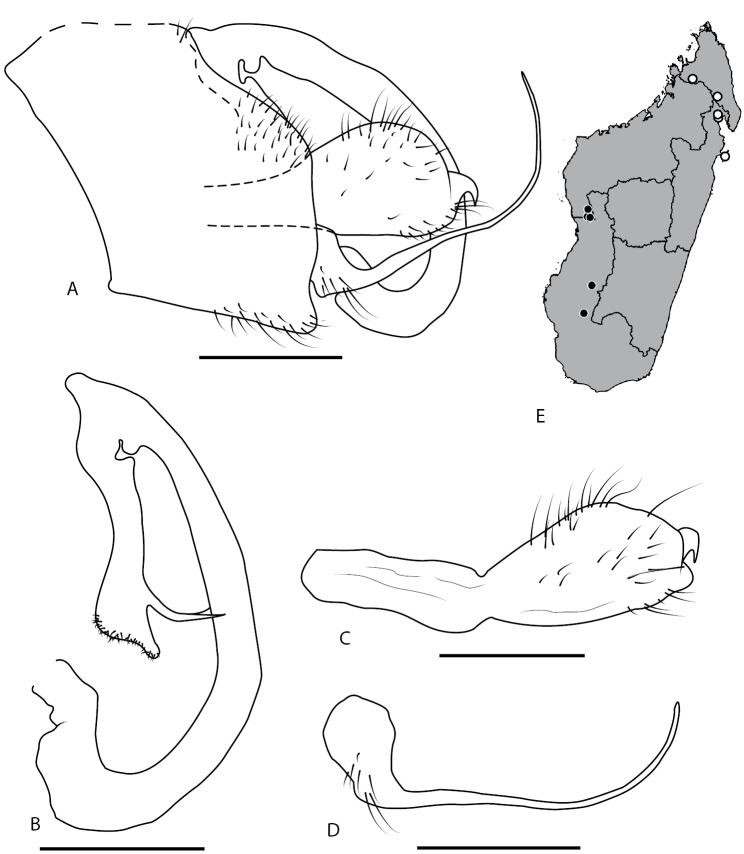
*Locris bipunctata atra* (Lallemand), male terminalia of the paratype, in lateral view (**A–D**) and *Locris bipunctata* (Signoret), distribution map (**E**) **A** pygofer, anal tube, aedeagus, left paramere and left subgenital plate **B** aedeagus **C** left paramere **D** left subgenital plate **E** black dots: occurrences for *L. bipunctata atra*, white dots: occurrences for *L. bipunctata bipunctata*. Scale bars: 1 mm.

When Lallemand (1950) described *Locris bipunctata atra*, he described it as a variety, which according to the article 45.6.4 of the International Code of Zoological Nomenclature (ICZN 1999) should now be considered as a subspecies. Therefore, *Locris bipunctata* now contains two subspecies, easily distinguished from each other by the colouration of their tegmina, red for *L. b. bipunctata* (Fig. 1) and nearly completely black for *L. b. atra* (Fig. 2), the latter appearing as a strong melanisation. Geographically, the two subspecies are clearly separated (Fig. 3E) but from the material examined, no difference can be observed in the male terminalia between the two subspecies. For this reason, we decided to keep them as subspecies and illustrate here only the male terminalia of the paratype specimen of the subspecies *L. bipunctata atra* (Fig. 3A–D).

#### Distribution.

Madagascar (Fig. 3E), *L. bipunctata bipunctata* in northern Madagascar, *L. bipunctata atra* in western Madagascar.

#### Description of the male terminalia

(Fig. 3A–D). Pygofer (Fig. 3A), in lateral view almost trapezoidal with posterior margin being the longest side and slightly cut out in its first dorsal third. Aedeagus consists of two parts, first basal part, representing 2/3 of total length, tubular and almost same thickness over its entire length, curving regularly down then dorsally, and bearing on its apical dorsal part a small hump directed dorso-anteriorly; second part, hanging from first, narrowing in its middle then developing posteriorly a pair of pointed processes and finishing in a smooth and flattened apex slightly digit shaped ventro-posteriorly (Fig. 3B). Parameres smoothly widening from pygofer’s attachment and finishing posteriorly by a curved spine (Fig. 3C). Subgenital plates large at base, narrowing quickly in a long and fine structure curving gently dorsally on its apical half (Fig. 3D).

Body length: 7.5–10 mm.

#### Material examined.

Nominotypical subspecies–10 males. [Ambabalame, Madagascar], [Coll. Mus. Congo, Coll. V. Lallemand], [♂], [R. Det., 6664 H], [Muséum Paris, MNHN (EH) 24628]; [Madagascar], [Lavaudon], [Collectic E de Bergev], [Muséum Paris], [Muséum Paris, MNHN (EH) 24115], [Muséum Paris, MNHN (EH) 24625]; [Madagascar Nord, distr. d’Ambanja, N. de Beangona-Ambevy, Vallée d’Antremade, 400 m, II-1964, P. Soga], [Museum Paris]; [Museum Paris, Sainte-Marie-de-Madagascar, Coll. Noualhier, 1898]; [Museum Paris, Madagascar, Baie d’Antongil, A. Mocquerys, Coll. Noualhier, 1898], [Muséum Paris, MNHN (EH), 24720]; [Museum Paris, Madagascar, Baie d’Antongil, A. Mocquerys, Coll. Noualhier, 1898], [*Locris bipunctata* Sign.], [Muséum Paris, MNHN (EH), 24722]; [Madagascar Nord, distr. d’Ambanja, N. de Beangona-Ambevy, Vallée d’Antremabe, 400, II – 1964, P. Songa], [Muséum Paris], [Muséum Paris, MNHN (EH), 24719]; [Coll. Mus. Tervuren, Madagascar: Fampanambo, 1962, J. Vadon], [*Locris bipunctata* Signoret, H. Synave det., 1965], [*Locris bipunctata* Sign ♂♂], [Muséum Paris, MNHN (EH), 24721]; [Madagascar Amber-Geb.], [*Locris vicina* Sign. ♂ Edm. Schmidt determ. 1911], [Miz Pan Warszawa 12/1945, 2487]; [Madagascar Amber-Geb.], [*Locris vicina* Sign. ♂ Edm. Schmidt determ. 1911], [970], [Miz Pan Warszawa. 12/1945, 2488]; and [Madagascar Amber-Geb.], [*Locris vicina* Sign. ♂ Edm. Schmidt determ. 1911], [Miz Pan Warszawa. 12/1945, 2489].

Subspecies atra – ***Paratype*** (male). [Entre Ambakaka et Bekopaka sur la Manambolo, Inst. Scient. Madagascar, VII – 49, RP], [Muséum Paris], [Paratype], [Muséum Paris MNHN (EH) 1474], [*Locris bipunctata v. atra* Lall.], [Muséum Paris MNHN (EH) 24634] – 5 males. [CASENT3004533], [Madagascar: Mahajanga, Prov: Parc National Tsingy de Bemaraha, 3.4 km 93°E, Bekopaka, Tombeau Vazimba, Elev 50 m, 6–10 Nov. 2001], [19°8'31"S, 44°49'41"E, coll: Fischer, Griswold et al., California Acad. Of Sciences malaise trap, in tropical dry forest, coll. Code: BLF4233], [♂], [*Locris bipunctata atra* Lallemand, 1950, A. Soulier-Perkins det 2018]; [Madagascar Ouest, S-P. Antsalova Antsingy, Rés. Nat. 9, A. Peyrieras, I-1975], [Museum Paris]; [Madagascar Lambomakandro, Tuléar], [Museum Paris, 1935, B. Catala], [H. Synave det., 1979, Locris bipunctata var. atra Lall.]; [Andobo 190 m, forêt Antsingy, det. Antsalova, II – 57, P. Guiv], [Institut Scientifique, Madagascar], [Muséum Paris, MNHN (EH), 24718]; and [Madagascar, Lambomakandro, Tuléar], [Muséum Paris, 1935, R. Catala], [H. Synave., 1979, *Locris bipunctata var. atra* Stall.], [Muséum Paris, MNHN (EH), 24717]; 2 females. [♀], [Madagascar, province de Toliara, massif du Makay, 159 m, 21°40'29.4"S, 44°59'36.2"E], [Muséum Paris, ft Ambalamanga, rv Mangoky, 18-I-2011, A. Soulier-Perkins rec.], [*Locris bipunctata atra* Lallemand, 1950, A. Soulier-Perkins det. 2020], [Muséum Paris, MNHN (EH), 24759]; [♀], [Madagascar, province de Toliara, massif du Makay, 159 m, 21°40'29.4"S, 44°59'36.2"E], [Muséum Paris, Près rivière, PL, 20-I-2011, A. Soulier-Perkins rec.], [*Locris bipunctata atra* Lallemand, 1950, A. Soulier-Perkins det. 2020], [Muséum Paris, MNHN (EH), 24760].

### Locris nigrolimbata

Taxon classificationAnimaliaHemipteraCercopidae

(Lallemand, 1910) comb. nov.

755EF0FD-9DBF-5338-877E-D22EEC802D64

Figures 4 , 5

Locris johannae var. nigrolimbata Lallemand, 1910: 47 (description)

#### Note.

**Figure 4.**
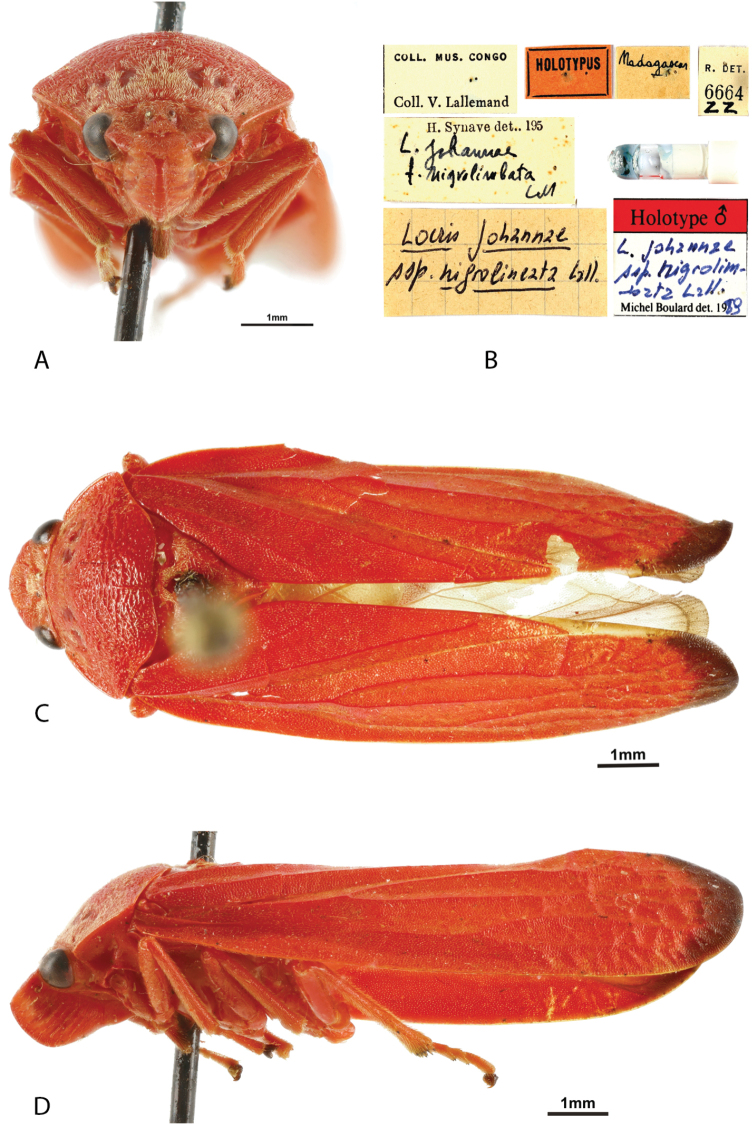
*Locris nigrolimbata* (Lallemand), male holotype **A** frontal view **B** labels **C** dorsal view **D** lateral view.

When Lallemand (1910) described the species *Locris johannae* (from the southwest bank of Lake Tanganyika) he described as well a variety *nigrolimbata* from Madagascar. According to article 45.6.4 of the International Code of Zoological Nomenclature (ICZN 1999), *Locris johannae nigrolimbata* should be regarded as a subspecies (Fig. 4). This subspecies supposedly differs by the colouration of the tegmina. *Locris j. johannae* has completely red tegmina while *L. j. nigrolimbata* bears some black at their apex (Lallemand 1910). Our studies of material from Tanzania and Kenya, showed that the specimens with the black apex tegmina have the same male genitalia as the holotype of *L. j. nigrolimbata* while the specimens with the entire red tegmina have different male genitalia. This led us to consider *L. j. nigrolimbata* as a valid species and here change its rank to *L. nigrolimbata* comb. nov. However, we remain for now with only one specimen of this species from Madagascar, the other specimens were collected on the African continent.

#### Distribution.

Madagascar and eastern Africa

#### Description of the male terminalia

**Figure 5.**
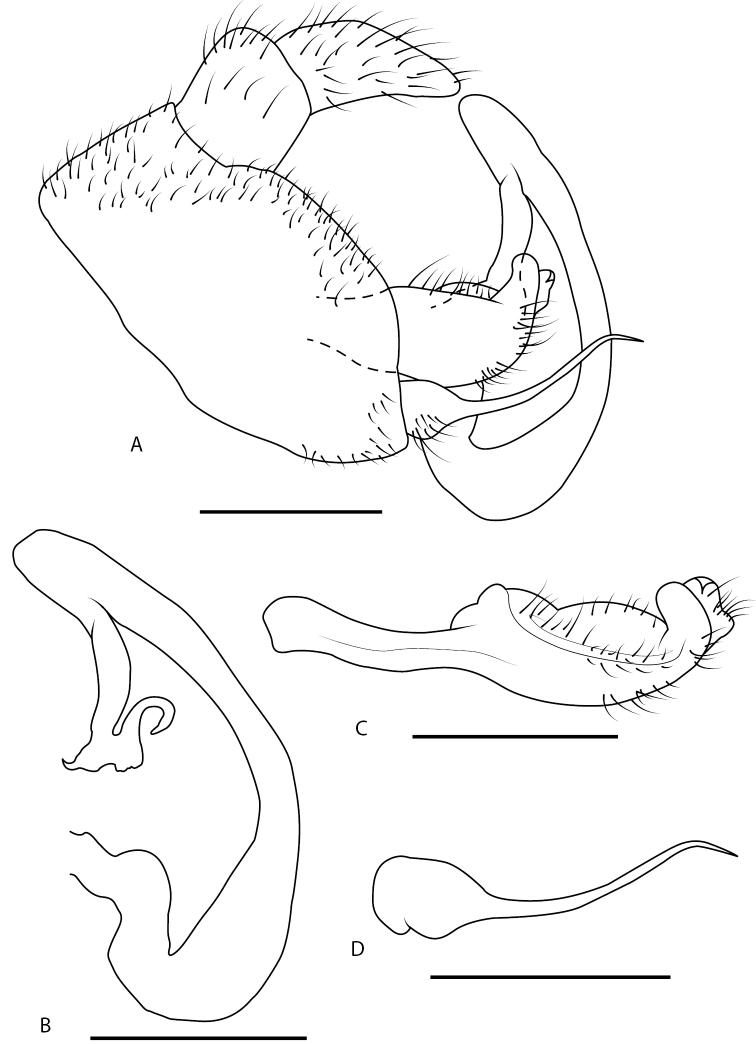
*Locris nigrolimbata* (Lallemand), male terminalia, in lateral view **A** pygofer, anal tube, aedeagus, left paramere and left subgenital plate **B** aedeagus **C** left paramere in latero-dorsal view **D** left subgenital plate. Scale bars: 1 mm.

**(Fig. 5).** Pygofer (Fig. 5A), in lateral view, dorsal margin straight and perpendicular to anterior margin, posterior margin generally convex. Aedeagus consist of two parts, first basal part, representing 3/4 of total length with a base elbow shaped before widening ventrally then curving up dorsally and narrowing into a tubular structure almost of same thickness to its regular rounded dorsal apex; second part, hanging from first, its width is regular for most of its length with apex in shape of a swan, neck oriented posteriorly (Fig. 5B). Parameres prolonged apically by two little structures, inner one curved into a spine and external one rounded (Fig. 5C). Subgenital plates wide at base then abruptly narrowing in a long filament shape curved downward at its apex (Fig. 5D).

Body length: 8–10.5 mm.

#### Material examined.

***Holotype*** (male). [Holotypus], [Madagascar], [Coll. Mus. Congo, Coll. V. Lallemand], [R. DET. 6664 z z], [Locris johannae ssp. nigrolimbata Lall.], [H. Synave det.. 195 L. johannae f. nigrolimbata Lall.], [Holotype ♂ L. johannae ssp. nigrolimbata Lall. Michel Boulard det. 1989] – 4 males. [Afrique orient. Anglaise Voï Alluaud & Jeannel Mars 1911 . 600 m . St. 60], [Coll. Mus. Congo Coll. V. Lallemand], [R. Det. 6665]; [Afr. Or. Angl. (Wa-Kikuyu), Fort-Hall Alluaud & Jeannel Janv. 1912 – 1330 m – St. 80], [Coll. Mus. Congo Coll. V. Lallemand], [R. Det. 6665]; [♂], [Nairobi B. E. A.], [G. Babault, avril 1923], [*Locris johannae* ssn. *nigrolimbata*], [Muséum Paris, MNHN (EH), 24629]; and [Tanzanie: Mts Uluguru, Kimboza ofr. Héliophile, alt. 600M 24-30/VII/71], [Coll. Mus. Tervuren, Mission Mts. Uluguru, L. Berger, N. Leleup, J. Debecker V/VIII/71], [H. Synave det. 1957, *Locris johannae* Lall.], [Muséum Paris, MNHN (EH), 24635].

### Locris vicina

Taxon classificationAnimaliaHemipteraCercopidae

(Signoret, 1860)

91E7A29E-F630-5C33-8AA6-7E80FF872994

Figures 6 , 7

Monecphora vicina Signoret, 1860: 182 (original description)
Locris vicina : Stål 1866: 60 (transfer).

#### Distribution.

Madagascar (Fig. 7E)

#### Description of the male terminalia

(Fig. 7A–D). Pygofer (Fig. 7A) in lateral view, dorsal and anterior margins perpendicular, posterior margin S-shaped and making an acute angle with the ventral margin. Aedeagus consists of two parts, first basal part, representing a small 2/3 of total length with a very regular tubular shape almost curving as half a circle, its dorsal apical part bearing two little bumps oriented dorso-anteriorly; second part is hanging from first, it is widening smoothly, two processes are pointing posteriorly at mid-length, apex bulbous and covered in a dense padding (Fig. 7B), genital duct passes through aedeagus and open in the centre of padded apex (Fig. 7B). Parameres with a dorsal margin S-shaped giving it a cup shape prolonged apically by a spine curved downward (Fig. 7C). Subgenital plates wide at base and narrowing abruptly in a long filament structure curving abruptly dorsally on last third (Fig. 7D).

**Figure 6.**
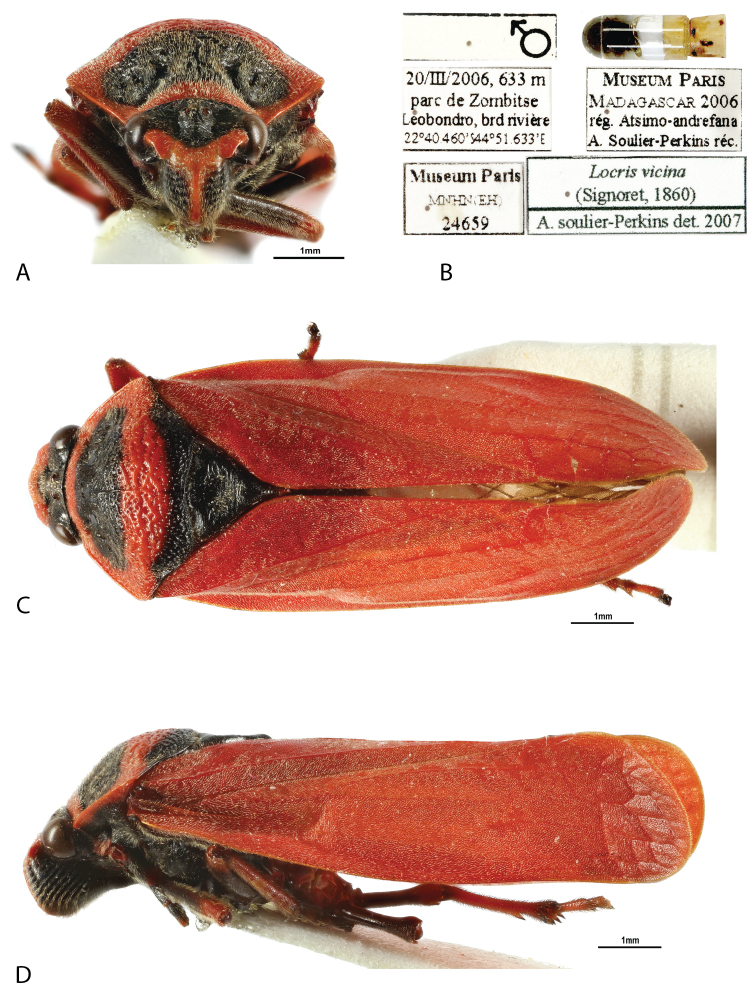
*Locris vicina* (Signoret, 1860), male **A** frontal view **B** labels **C** dorsal view **D** lateral view.

**Figure 7.**
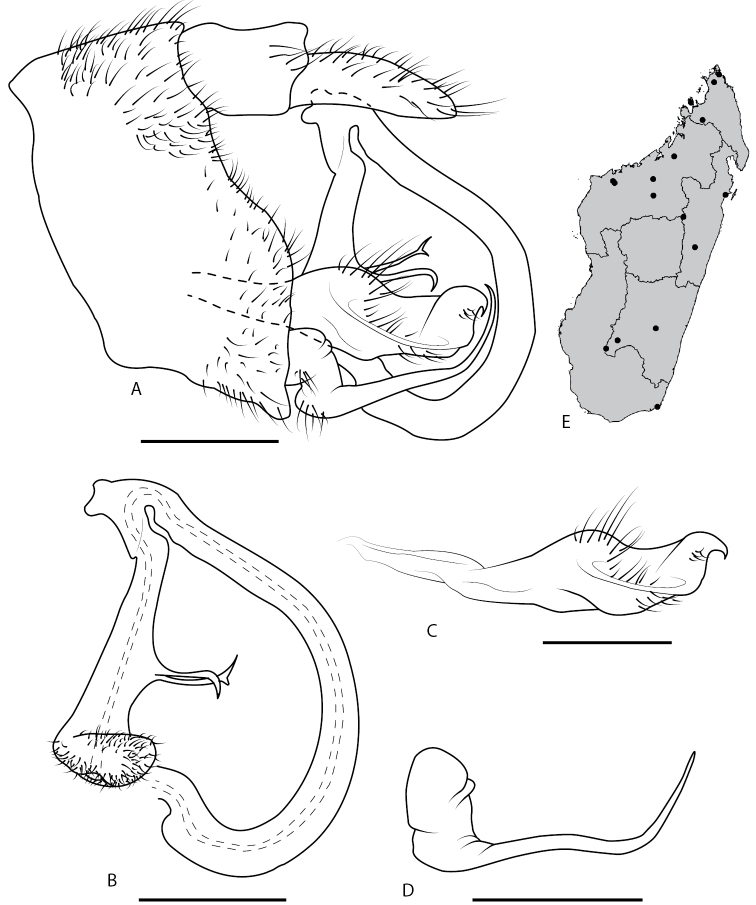
*Locris vicina* (Signoret, 1860), male terminalia, in lateral view and distribution map **A** pygofer, anal tube, aedeagus, left paramere and left subgenital plate **B** aedeagus **C** left paramere **D** left subgenital **E** distribution map. Scale bars: 1 mm.

Body length: 10.5–12.5 mm.

#### Material examined.

MNHN Collection–1 female [♀], [Madagascar. Tam.], [Soanierana-Ivongo, 8 . XI . 57, F. Keiser], [R. I. Sc. N. B., I. G. 23.285], [Muséum Paris, MNHN (EH), 24723]; –10 males. [20/III/2006, 633 m, parc de Zombitse Leobondro, brd rivière, 22°40.460'S, 44°51.633'E], [Muséum Paris, Madagascar 2006, rég. Atsimo-andrefana, A. Soulier-Perkins réc.], [*Locris vicina* (Signoret, 1860), A. Soulier-Perkins det. 2007], [♂], [Muséum Paris, MNHN (EH), 24659]; [♂], [Museum Paris Madagascar, Antanambé, Mocquerys, Coll.Noualhier 1898]; [♂], [Museum Paris, Madagascar, catat 1844-91]; [Madagascar Nord, distr. d’Ambanja, N. de Beangona-Ambevy, Vallée d’Antremabe, 400 m, II – 1964, P. Soga], [Muéum Paris], [Muséum Paris, MNHN (EH), 24724]; [Fort. Dauphin], [Muséum Paris, 1933, A. Seyrig], [Muséum Paris, MNHN (EH), 24725]; [Maeyatanana, Madagscar], [Collection le Moult, Naturaliste. Paris], [R. Mus. Hist. Nat., Belg. I. G. 12.595], [Muséum Paris, MNHN (EH), 24726]; [Madagascar, province de Mahajanga, P N Tsingy de Namoroka, 16°28'08"S, 45°20'52"E], [Muséum Paris, 09-IX-2012, Th. Bourgoin rec.], [Muséum Paris, MNHN (EH), 24727]; [21/XI/2005, région lac Alaotra, bord riv. Mavolava, 17°40.357'S, 47°54.289'E], [904 m, entre Ambakireny et Morano-Chrome], [Muséum Paris, Madagascar 2005, Bourgoin, Ouvrard, Attié, Soulier-Perkins], [*Locris vicina* (Signoret, 1860), A. Soulier-Perkins det. 2007], [♂], [Muséum Paris, MNHN (EH), 24728]; [Madagascar Mahajanga, P.N. Tsingy de Namoroka, wetland near village, 16°23'50"S, 45°17'12"E], [Muséum Paris, 29-X-2016, PL., T. Bourgoin, G. Kunz & A. Soulier-Perkins rec.], [Muséum Paris, MNHN (EH), 24729] – RIScNB [Ampijoro, Tsaramandroso], [Institut scientifique, Madagascar], [H. Synave det., 1957, *Locris vicina* Signoret], [R. I. Sc. N. B., I. G. 21.002]; MIIZ collection – 5 males [Madagasckar, Ambodimanga, Hammerstem S., I. 1906], [*Locris bipunctata* Sign. ♂ Edm. Schmidt determ. 1909], [Miz Pan Warszawa. 12/1945, 2490]; [Madagaskar, Ambodimanga, Hammerstem S., I. 1906], [*Locris bipunctata* Sign. ♂ Edm. Schmidt determ. 1909], [Miz Pan Warszawa. 12/1945, 2491]; [Madagaskar, Ambodimanga, Hammerstem S., I - II. 1906], [*Locris bipunctata* Sign. ♂ Edm. Schmidt determ. 1909], [Miz Pan Warszawa. 12/1945, 2492]; [Madagaskar, Ambodimanga, Hammerstem S., I - II. 1906], [*Locris bipunctata* Sign. ♂ Edm. Schmidt determ. 1909], [Miz Pan Warszawa. 12/1945, 2493]; and [Madagaskar, Ambodimanga, Hammerstem S., I - II. 1906], [*Locris bipunctata* Sign. ♂ Edm. Schmidt determ. 1909], [Miz Pan Warszawa. 12/1945, 2494].

Casent collection – 7 males [CASENT8107240], [Madagascar: Majunga, Ampijoroa National Park, 160 km N of Maevatanana, on RN 04, elev 43 m, 25 Jan–7 Feb 2005], [16°19.16'S, 46°48.80'E, California Acad of Sciences, coll: M. Irwin, R. Harin’Hala, malaise trap – in deciduous forest, MA-25-42]; [CASENT8107233], [Madagascar: Majunga, Ampijoroa National Park, 160 km N of Maevatanan, on RN 04, elev 43 m, 2–9 November 2003], [16°19.16'S, 46°48.80'E, California Acad of Sciences, coll: M. Irwin, R. Harin’Hala, malaise trap – in deciduous forest, MA-25-22]; [CASENT8107236], [Madagascar: Majunga, Ampijoroa National Park, 160 km N of Maevatanan, on RN 04, elev 43 m, 10–21 January 2004], [16°19.16'S, 46°48.80'E, California Acad of Sciences, coll: M. Irwin, R. Harin’Hala, malaise trap – in deciduous forest, MA-25-29]; [CASENT8077112], [Perinet, Madagascar, 14 Jul. 1966, Liusnan], [*Locris vicina*, det. Penny, 99]; [CASENT8077114], [Madagascar, Hellville, Nossi-Be Isl., XI – 18 –1959], [E. S. Ross, Collector], [*Locris vicina*]; [CASENT3002067], [Madagscar: Province, d’Antsiranana, Montagne des Français. 7.2 km 142°SE, Antsiranana (=Diego Suarez), Elev 180 m, 22–28 Feb 2001], [12°19'22°S, 49°20'17°E, colls: Fisher. Griswold et al. Calif. Academy of Sciences, malaise trap, in tropical dry forest. Code: BLF3130]; and [CASENT3008146], [Madagascar: Fianarantsoa, Province, Parc National d’Isalo, 9.1 km 354°N Ranohira, elev 725 m, 27–31 Jan 2003, 22°28'54"S, 045°27'42"E], [coll. Fisher, Griswold et al. California Acad. Of Sciences, collected at light-gallery forest, collection code: BLF7304].

### Identification key to the species and subspecies of *Locris* from Madagascar

**Table:** 

1	Pronotum bearing two red dots in middle	**2**
–	Pronotum not bearing two red dots in middle	**3**
2	Tegmina completely red (Fig. 1)	***Locris bipunctata bipunctata* (Signoret)**
–	Tegmina black except for red anal margin (Fig. 2)	***Locris bipunctata atra* Lallemand**
3	Tegmina completely red (Fig. 6)	***Locris vicina* (Signoret)**
–	Tegmina red with black apex (Fig. 4)	***Locris nigrolimbata* (Lallemand) comb. nov.**

### Bourgoinrana

Taxon classificationAnimaliaHemipteraCercopidae

Soulier-Perkins, 2012

575DB6A6-05AB-568F-8CD4-F59B134827B7

#### Type species.

*Amberana perinetana* Synave, 1957.

### Bourgoinrana beondrokaensis

Taxon classificationAnimaliaHemipteraCercopidae

Le Cesne & Soulier-Perkins sp. nov.

0D3AF096-430D-5011-8E26-907C30E8D0C7

http://zoobank.org/75FDC14D-6616-43C9-AE85-EA7135771F4A

Figures 8 , 9

#### Diagnosis.

Uniformly coloured brownish with smokey yellowish tegmina compared to the similar *B. sandrangatensis* which has a red head, thorax and base of tegmina. It also differs from this species by the length of its subgenital plates, 1.18 times longer than its pygofer height compared to 1.48 for *B. sandrangatensis*.

#### Distribution.

Mount Beondroka in the natural reserve of Marojejy, Madagascar (Fig. 9E).

#### Description.

Total length of male holotype 7.9 mm (tegmina included), paratypes 8.4 and 8.6 mm. Flattened ventro-dorsally. Head in dorsal view, 1.6 times wider between eyes than long in midline, anterior and posterior margins gently and regularly curved. Ocelli very close to each other with distance between eye and ocellus 9 times greater than between ocelli, located close to head posterior margin. Pronotum slightly convex, 1.8 times wider than long in midline, posterior margin wave-shaped, concave in middle. Tegmina 3.8 times longer than wide, M and CuA with a common stem at base and forking around 1/3 of tegmen length, ScP+R(+MA) forking after mid-length of tegmen, M and CuA both forking in apical third of tegmen. Metatibia bearing 1 lateral spine at 2/3 of its length.

#### Description of the male terminalia

**(Fig. 9A–D).** Pygofer height 1.6 mm (Fig. 9A) in lateral view, dorsal margin almost straight, posterior margin almost straight 2/3 of its dorsal length then curving strongly anteriorly before having a final straight section, ventral margin straight and shorter than dorsal margin, anterior margin S-shaped. Subgenital plates 1.18 longer than pygofer height, gently and regularly curving dorsally with a rounded apex pointing dorso-posteriorly, thickness gradually diminishing from base to apex with a slight constriction before apex. Parameres in lateral view with a small hump in the middle of dorsal margin ending in a narrow elongate finger-shape apex slightly curved inward (Fig. 9C, D), ventral margin ending in a finger-shape apex shorter and less narrow than dorsal one; in dorsal view, internal margin cut out in two small extension facing each other (Fig. 9C). Lateral plates present with dorsal margin straight, making a rounded acute angle with the postero-ventral margin. Aedeagus long, shaped as a circumvented tube with a thumb-shaped extension on its dorsal margin, oriented dorso-anteriorly, small constriction before thumb extension; apical part bifid, prolonging ventral margin a lateral toothed extension folding posteriorly with apex pointing antero-ventrally and prolonging dorsal margin a thin ending in a lanceolate shape pointing anteriorly (Fig. 9B).

#### Colouration.

**Figure 8.**
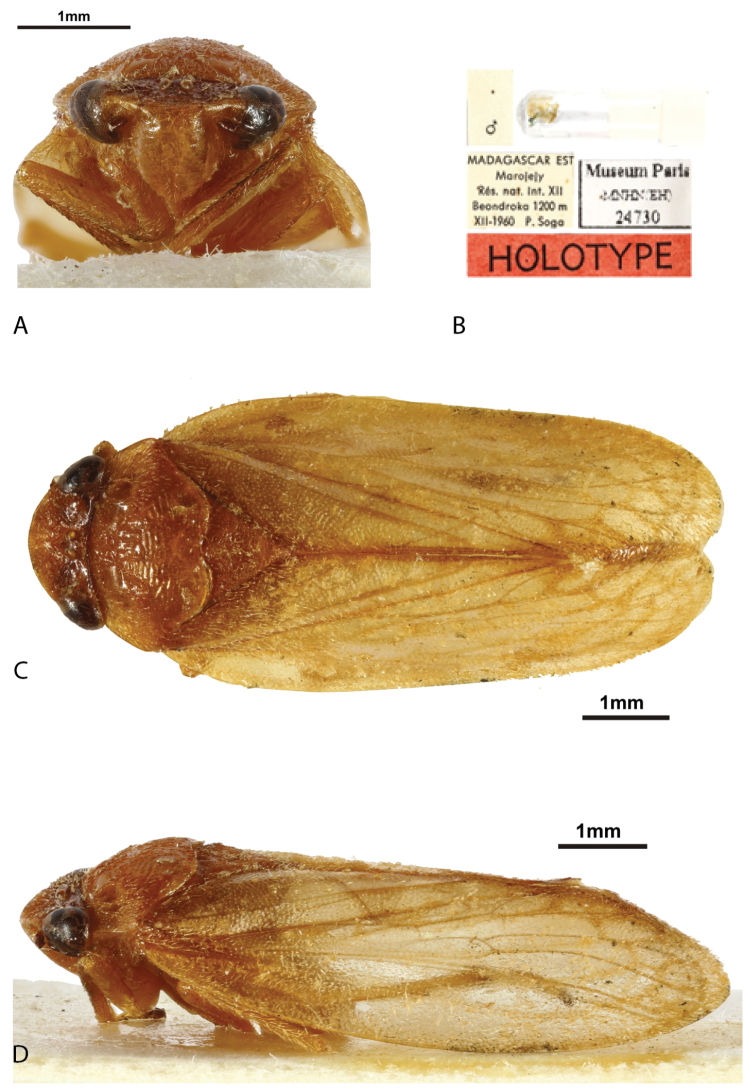
*Bourgoinrana beondrokaensis* Le Cesne and Soulier-Perkins, sp. nov., male **A** frontal view **B** labels **C** dorsal view **D** lateral view.

Generally yellowish brown, head darker between eyes along posterior margin, pedicel of antennae dark brown. Tegmina smokey yellow and translucent. Abdomen red and legs yellowish with darker tarsal segments, lateral metatibial spine black (Fig. 8).

#### Etymology.

The species is named after the type locality, Beondroka.

**Figure 9.**
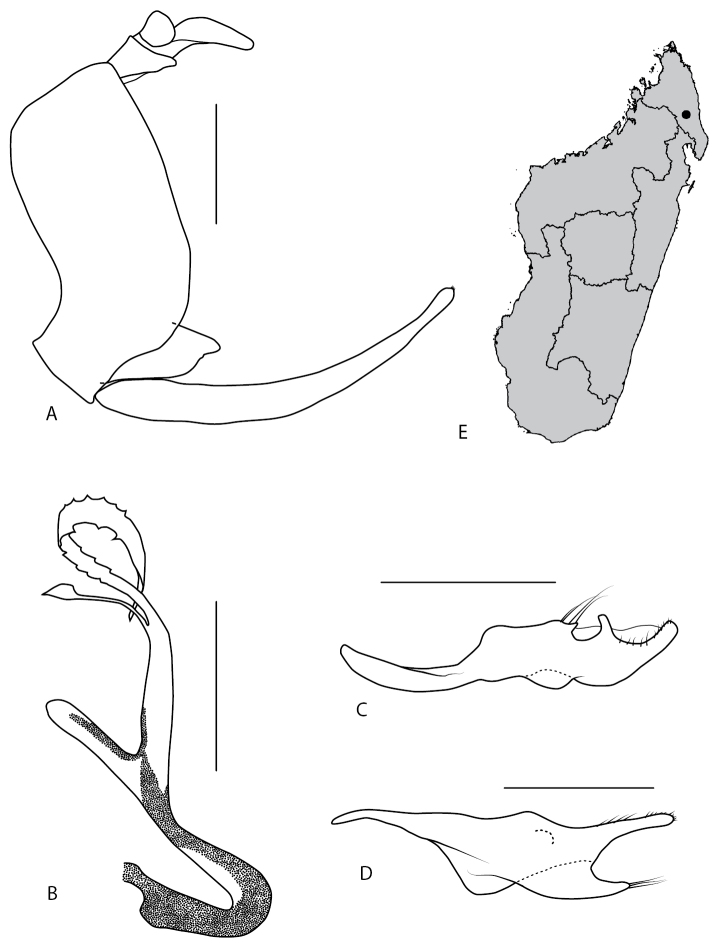
*Bourgoinrana beondrokaensis* Le Cesne and Soulier-Perkins sp. nov., male terminalia and distribution map **A** pygofer, anal tube, left lateral plate and left subgenital plate **B** aedeagus **C** left paramere in lateral view **D** left paramere, in dorsal view **E** distribution map. Scale bars = 0.5 mm.

#### Type locality.

Madagascar Est, Marojejy, Rés. Nat. Int. XII, Beondroka 1200 m, XII.1960, P. Soga

#### Type material.

***Holotype*** (male), pinned: [Madagascar Est, Marojejy, Rés. Nat. Int. XII, Beondroka 1200 m, XII.1960, P. Soga], [♂], [Holotype], [Museum Paris, MNHN(EH) 24730]**. *Paratypes***: 1 male: [Madagascar Est, Marojejy, Rés. Nat. Int. XII, Beondroka 1200 m, XII.1960, P. Soga], [Museum Paris], [Paratype], [Museum Paris, MNHN(EH) 24731] 5 females: [Madagascar Est, Marojejy, Rés. Nat. Int. XII, Beondroka 1200 m, XII.1960, P. Soga], [♀], [Paratype], [Museum Paris, MNHN(EH) 24732]; [Madagascar Est, Marojejy, Rés. Nat. Int. XII, Beondroka 1200 m, XII.1960, P. Soga], [♀], [Paratype], [Museum Paris, MNHN(EH) 24734]; [Madagascar Est, Marojejy, Rés. Nat. Int. XII, Beondroka 1200 m, XII.1960, P. Soga], [♀], [Paratype], [Museum Paris, MNHN(EH) 24735]; [Madagascar Est, Marojejy, Rés. Nat. Int. XII, Beondroka 1200 m, XII.1960, P. Soga], [♀], [Paratype], [Museum Paris, MNHN(EH) 24736]; and [Madagascar Est, Marojejy, Rés. Nat. Int. XII, Beondroka 1200 m, XII.1960, P. Soga], [Museum Paris], [Paratype], [Museum Paris, MNHN(EH) 24733]

### Identification key to the species of *Bourgoinrana*

**Table:** 

1	Pronotum uniformly coloured	**2**
–	Pronotum with an anterior transverse yellowish band	***B. perinetana* (Synave)**
2	Tegmina completely red	***B. rubescens* (Synave)**
–	Tegmina mostly smokey yellowish translucent	**3**
3	Head, thorax and base of tegmina red	***B. sandrangatensis* (Synave)**
–	Head and thorax ochre-brown and tegmina entirely smokey yellowish	***B. beondrokaensis* Le Cesne & Soulier-Perkins sp. nov.**

## Supplementary Material

XML Treatment for Locris

XML Treatment for Locris bipunctata

XML Treatment for Locris nigrolimbata

XML Treatment for Locris vicina

XML Treatment for Bourgoinrana

XML Treatment for Bourgoinrana beondrokaensis
